# SOCIAL NETWORKS AND NEIGHBORHOOD SATISFACTION OF AFRICAN AMERICAN OLDER ADULTS: AN ATLANTA STUDY OF RELOCATION

**DOI:** 10.1093/geroni/igz038.616

**Published:** 2019-11-08

**Authors:** Nia Reed

**Affiliations:** Georgia State University, Atlanta, Georgia, United States

## Abstract

Atlanta was the first major city to offer federally-funded public housing and it is one of the first to demolish it. Unlike other cities undergoing public housing transformation through demolition under Housing for People Everywhere Program (HOPE VI), the Atlanta Housing Authority (AHA) targeted senior housing as part of the demolition process. Investigators conducting the Urban Health Initiative (UHI) study collected three waves of data (baseline, 6-month post-relocation, and 24-month post-relocation) from relocated seniors and a comparison group of seniors who aged-in-place. To understand the interactions between public housing residents and varied components of their environments, including social networks and neighborhood satisfaction, I will use place attachment theory to frame my research, as sense of place is rooted within the interplay of community cultural wealth components. I will also use aging-in-place theory, which refers to individuals’ ability to grow old in their own homes and communities, while adjusting to needed modifications associated with aging and mobility. Analysis of Covariance will be applied to understand the relationship between social networks, relocation, and neighborhood satisfaction among older adults who age-in-place, compared to those who relocated.

